# Functional Diversification of Paralogous Transcription Factors via Divergence in DNA Binding Site Motif and in Expression

**DOI:** 10.1371/journal.pone.0002345

**Published:** 2008-06-04

**Authors:** Larry N. Singh, Sridhar Hannenhalli

**Affiliations:** Penn Center for Bioinformatics, Department of Genetics, University of Pennsylvania, Philadephia, Pennsylvania, United States of America; The Wellcome Trust Sanger Institute, United Kingdom

## Abstract

**Background:**

Gene duplication is a major driver of evolutionary innovation as it allows for an organism to elaborate its existing biological functions via specialization or diversification of initially redundant gene paralogs. Gene function can diversify in several ways. Transcription factor gene paralogs in particular, can diversify either by changes in their tissue-specific expression pattern or by changes in the DNA binding site motif recognized by their protein product, which in turn alters their gene targets. The relationship between these two modes of functional diversification of transcription factor paralogs has not been previously investigated, and is essential for understanding adaptive evolution of transcription factor gene families.

**Findings:**

Based on a large set of human paralogous transcription factor pairs, we show that when the DNA binding site motifs of transcription factor paralogs are similar, the expressions of the genes that encode the paralogs have diverged, so in general, at most one of the paralogs is highly expressed in a tissue. Moreover, paralogs with diverged DNA binding site motifs tend to be diverged in their function. Conversely, two paralogs that are highly expressed in a tissue tend to have dissimilar DNA binding site motifs. We have also found that in general, within a paralogous family, tissue-specific decrease in gene expression is more frequent than what is expected by chance.

**Conclusions:**

While previous investigations of paralogous gene diversification have only considered coding sequence divergence, by explicitly quantifying divergence in DNA binding site motif, our work presents a new paradigm for investigating functional diversification. Consistent with evolutionary expectation, our quantitative analysis suggests that paralogous transcription factors have survived extinction in part, either through diversification of their DNA binding site motifs or through alterations in their tissue-specific expression levels.

## Introduction

Gene or even whole genome duplication provides the essential spare parts for evolutionary innovation[1]–[4]. Relaxed purifying selection immediately after duplication allows an organism to elaborate its existing functional repertoire through specialization and diversification of individual gene functions[5]–[8]. In particular, expansion of transcription factor (TF) gene families has played a substantial role in the evolution of organismal complexity by elaborating transcriptional networks[9]–[11].

**Figure 1:**
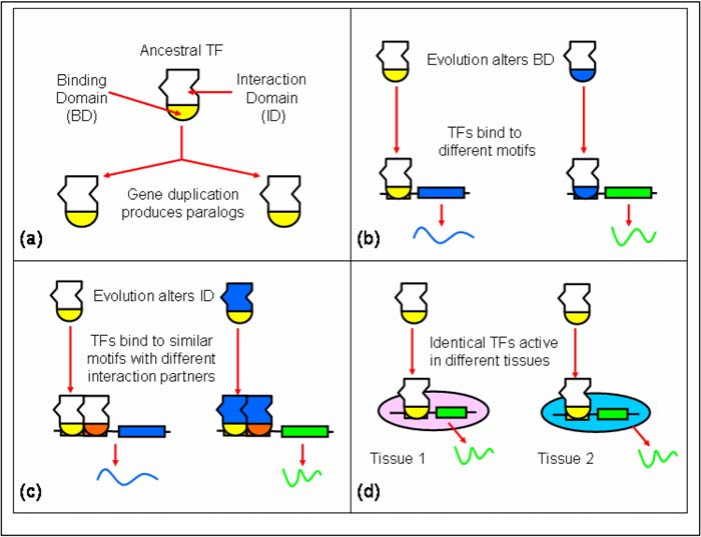
Evolutionary fates of TF paralogs. (a) Model of a TF showing the interaction domain (ID) and binding domain (BD). Duplication events produce two identical paralogous TFs. (b) As a result of evolutionary divergence of the BDs, the two paralogs bind to distinct DNA regulatory sites and thus, target distinct genes. (c) As a result of evolutionary divergence of the IDs, the two paralogs interact with distinct partners and thus, target distinct genes (or the same genes under different contexts) (d) As a result of evolutionary divergence of the expression patterns of the paralogous TF genes, the two TFs are active in different contexts, for instance, different tissues.

Paralogous genes (genes within a species evolutionarily-related by gene duplication events) that survive extinction often diversify by either assuming an entirely novel function, or specializing in some aspects of their original function while losing other functions[2]–[8]. However, in some cases an increased dosage is advantageous and the gene copies remain relatively conserved, partly mediated by gene conversion[12], [13]. The initially identical gene copies may functionally diversify *via* several distinct pathways. Figure 1 illustrates three possibilities that apply to TF genes in particular. First, the DNA binding domain may mutate, thereby altering the DNA binding site motif and hence the gene targets of the TF; this is common among paralogous TFs. Second, the interaction/activation domain may mutate thereby altering the TF's interacting partners[14]. Two paralogous TFs having similar DNA binding domains but with different interaction domains may result in one TF acting as a suppressor of its paralog by interfering with the sibling's binding; for example, Foxo1 and Foxa2 have highly similar DNA binding specificities but Foxo1 acts as a suppressor of Foxa2-mediated regulation of Pdx1 in pancreatic β-cells[15]. A third possible fate of duplicated TFs is the divergence of their gene expression[3]. Transcription factor Pax2, an essential regulator of nephrogenesis, regulates c-Ret specifically in kidney[16], while the paralog Pax3, with a similar DNA binding specificity, regulates the same gene in neural crest[17]. The relationship between these distinct pathways of functional diversification of paralogous TF genes is of fundamental interest from the perspective of understanding evolution of gene families, but has not been investigated previously.

Although mutations directly alter the genome, the resulting functional change is what drives evolutionary selection. A comparison of various diversification pathways in terms of their functional consequences is thus likely to reveal relationships among these pathways. For instance, mutations in the DNA binding domain of a TF gene are likely to alter the TF's DNA binding site motif. Availability of DNA binding site motifs of a vast number of human TFs[18], [19], as well as genome wide expression data[20] provides a novel opportunity to investigate the relationship between divergence in the DNA binding site motif and divergence in expression among paralogous TFs.

By analyzing a large collection of human paralogous TF pairs, we show for the first time that the paralogous pairs whose DNA binding site motifs are similar tend to have diverged expression patterns so that in any particular tissue at most one of the paralogs is expressed at a high level. Conversely, the paralogs that are highly expressed in a tissue tend to have dissimilar DNA binding site motifs. Our work represents a first attempt to quantify the biological expectation that paralogous TFs must diversify in one or more aspects of their function in order to survive extinction. We have extended our pair-wise analysis to TF families, demonstrating that in any given tissue there is a large separation in expression level between the family members with similar DNA binding site motifs. Furthermore, our finding is independent of the age of paralogs. TF paralogs with diverged binding site motifs tend to be diverged in their functions, as measured by GO terms. We also found that a decrease in tissue-specific gene expression is more frequent than what is expected by chance.

## Results

### Identifying human paralogous transcription factors

Using a stringent sequence similarity criteria as in [21], we identified 95 pairs of transcription factor (TF) gene paralogs for which there is a DNA binding site motif (Positional Weight Matrix or PWM) derived from human binding sites in the TRANSFAC database[18]. Our investigation of paralogous TFs is based on these 95 paralogous pairs corresponding to 98 unique genes; some genes pair with multiple distinct genes. Certain genes correspond to multiple transcripts, and in terms of transcripts, our data consists of 98 pairs with 123 unique transcripts. Certain transcripts correspond to multiple probe sets on the Affymetrix array. In terms of probe sets, our data consists of 390 probe set pairs with 201 unique probe sets.

### Human transcription factor paralogs that are expressed highly in a tissue tend to have distinct DNA binding site motifs and conversely, paralogs with similar DNA binding site motifs tend to be diverged in their tissue-specific expression

Consider two paralogous TF genes that are highly expressed in a particular tissue. If these two TFs recognize similar DNA binding site motifs then it is possible that these genes may interfere with each other's activity or they may potentially serve compensatory roles[22]. Another possibility is that the two TFs have dissimilar DNA binding site motifs and target different genes. The extent to which paralogous TFs that are highly expressed in the same tissues have diverged in their DNA binding site motifs is not known. Thus, for our set of paralogous TF pairs, we first tested whether there is correlation between the similarity of their DNA binding site motifs and their gene expression divergence.

Expression divergence for a pair of genes (*X*, *Y*) is commonly measured using the Pearson Correlation Coefficient (PCC) between *Ê_X_* and *Ê_Y_*, where *Ê_X_* and *Ê_Y_* represent the vectors of expressions for genes *X* and *Y*, respectively, in multiple tissues. Employing PCC as a measure of expression divergence yielded no significant correlation (Kendall's tau = 0.03 p value = 0.34) between expression similarity and DNA binding site motif similarity (Figure 1 in supplementary Data file S1). PCC only captures the pattern of expression across tissues and not the tissue-specific differences in expression, which is relevant for evolutionary selection. Any relationship between DNA binding site motif similarity and expression divergence is likely to be revealed in the context of individual tissues and is not evident if we consider overall gene profiles. To explore this hypothesis further, we directed our analysis towards tissue-specific expression divergence and its relationships to DNA binding site motif similarity.

**Figure 2:**
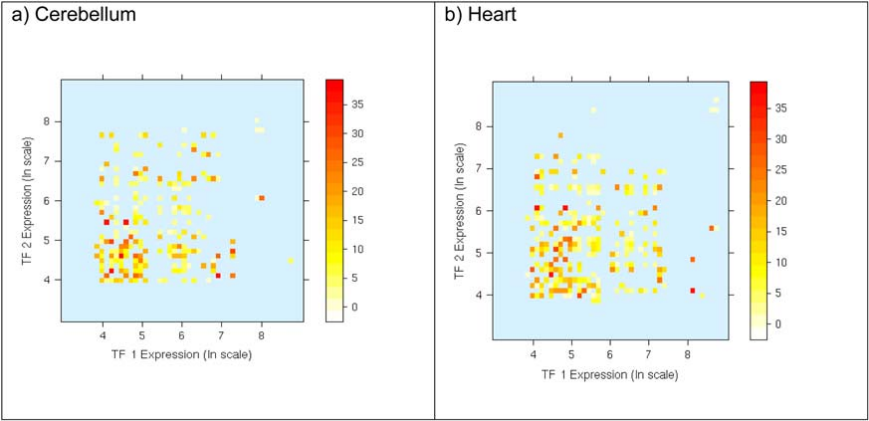
Paralogous TFs with high expression tend to have dissimilar DNA binding site motifs and conversely, paralogs with similar DNA binding site motifs tend to diverge in their tissue-specific expression. The expression values for each TF gene in a paralogous pair are indicated on the x and y-axes, and the value of the corresponding DNA binding site motif similarity is depicted by the color of the point. Binding site motif similarity of paralogous TFs is depicted by the color (darker color indicates higher binding site motif similarity) at a co-ordinate determined by the expression values (depicted in log scale) of the two paralogs (x and y-axes) (a) Using gene expression in Cerebellum (b) Using gene expression in Heart. We used log scale for expression to accommodate for extreme expression values.

**Figure 3:**
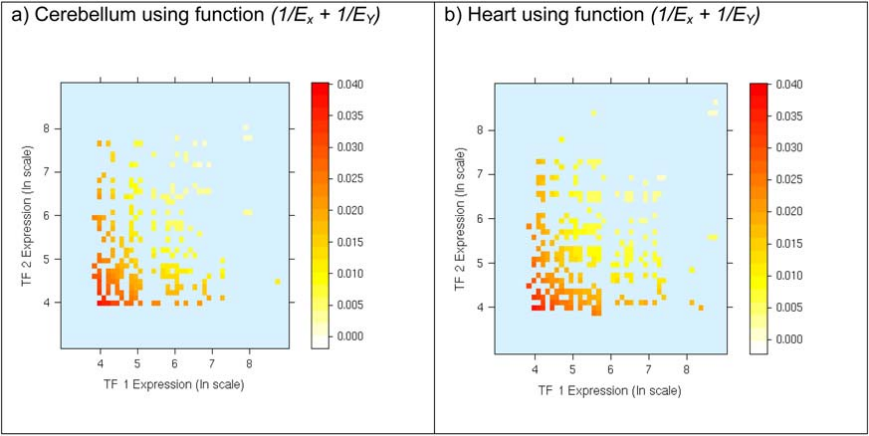
Measuring tissue-specific expression divergence. Theoretical plot of the function δE = (1/E_X_+1/E_Y_) where E_X_ and E_Y_ are expression levels of the two paralogs The expression values for each TF gene in a paralogous pair are indicated on the x and y-axes, and the value of the function δE is depicted by the color of the point (darker color indicates high δE). (a) Using gene expression in Cerebellum (b) Using gene expression in Heart. The function δE roughly approximates the trend for the DNA binding motif similarity in Figure 2. We used a log scale for expression to accommodate extreme expression values.

**Figure 4:**
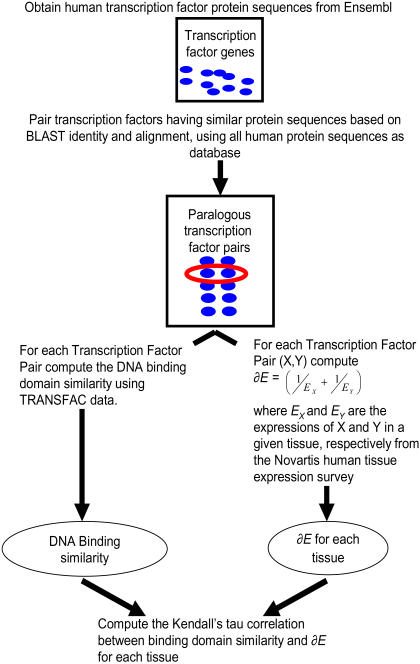
Schematic outlining the method for testing our hypothesis — correlation between expression divergence and DNA binding site motif similarity. For a set of human TF paralogs, and for a specific tissue, we estimate for each TF paralog, (i) tissue-specific expression (δE) and DNA binding site motif similarity (§B) (see text). Given these two values for each paralogous pair, we estimate their correlation using Kendall's tau measure and estimate the significance of correlation based on expression randomization.

Figures 2a and 2b depict for all TF paralogous pairs, the relationship between the tissue-specific expression of the two TF genes and their DNA binding site motif similarity in adult Cerebellum and Heart tissues, respectively. These plots indicate that for paralogous pairs with similar DNA binding site motifs, in general, at most one of the paralogs is expressed at a high level. Conversely, if both paralogs are highly expressed then their DNA binding site motifs tend to be dissimilar. In other words, the points corresponding to high DNA binding site motif similarity are mostly found near the origin (both paralogs have low expression) and near the axes (exactly one of the paralogs is highly expressed), and these points become increasingly sparse as we move away from the origin (both paralogs highly expressed). We found that this trend is consistent in all 79 tissues in Novartis dataset (data not shown). This observed dependence of DNA binding site motif similarity on the expression values of the two paralogs can be approximated by the function 
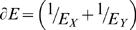
, where *E_X_* and *E_Y_* are the tissue-specific expression values of the two paralogs *X* and *Y*. Figures 3a and 3b show this theoretical dependence for the expression data in Cerebellum and Heart respectively. Thus, *∂E* is ‘high’ if either both TF genes have low expression or if exactly one of the TFs has high expression i.e., the two paralogs have diverged in their expression. We next quantify the correlation between DNA binding site motif similarity and *∂E* using the pipeline shown in Figure 4.

Given the expression data for a tissue, for each paralogous TF pair (*X*, *Y*), we computed *∂E* as defined above. We additionally computed the DNA binding site motif similarity *§B* between the PWMs corresponding to the two TFs using a previously benchmarked motif similarity measure based on the Pearson Correlation between PWM columns and Smith-Waterman un-gapped alignment of the PWMs[23]. Given the *∂E* and *§B* values for each paralogous TF pair, we computed the Kendall's tau rank correlation between *∂E* and *§B* values and estimated its significance based on 1000 permutations of the expression data. Using other measures of correlation such as Pearson or Spearman does not change the results (see methods and discussion). We repeated the *∂E∶§B* correlation analysis for each of the 79 human tissues from the Novartis dataset[20]. We found that in 48 of the 79 (61%) tissues there was a significant positive correlation (p-value≤0.05) between *∂E* and *§B*. However, there is a high degree of similarity among the expression profiles of related tissues and thus, the 79 correlation tests are not independent. Therefore, we considered a subset of 23 tissues that were deemed to be non-redundant by the authors of the Novartis dataset[24]. We found that in 17 of the 23 (74%) tissues there was a significant correlation (p-value≤0.05) between *∂E* and *§B*. At a 0.01 p-value threshold, 14 (61%) of the tissues show significance, and 9 (39%) of the tissues show significance at a 0.001 p-value threshold. The mean and standard deviation of the correlations (tau value) in the significant cases were 0.21 and 0.05, respectively. As a control, we repeated the above experiment after randomizing the expression data and found a significant correlation only in 4.5% of the tissues. By random chance, we expect 5% of the tissues to show significance at p-value threshold of 0.05. We also repeated our analysis using paralogous TF-pairs obtained from the KOG data (www.ncbi.nlm.nih.gov/COG/), for which we obtained 242 paralogous pairs with 99 unique genes. This analysis yielded 67.1% tissues significant at the 0.05 p-value threshold and 54.4% tissues at a 0.01 threshold corresponding to a 13- to 54-fold enrichment. Thus, our overall conclusions are robust across different definitions of paralog genes. We have performed a number of additional analyses to ensure the robustness of our general conclusions. These include (1) investigating the effect of excluding paralogous pairs with low expressions, (2) using alternative measures of expression divergence, and (3) using several way of aggregating multiple probe data and multiple PWMs. All these analyses yielded consistent results and the details are provided in Data file S1.

### Tissue-specific expression diversification of paralogous transcription factor genes

**Figure 5:**
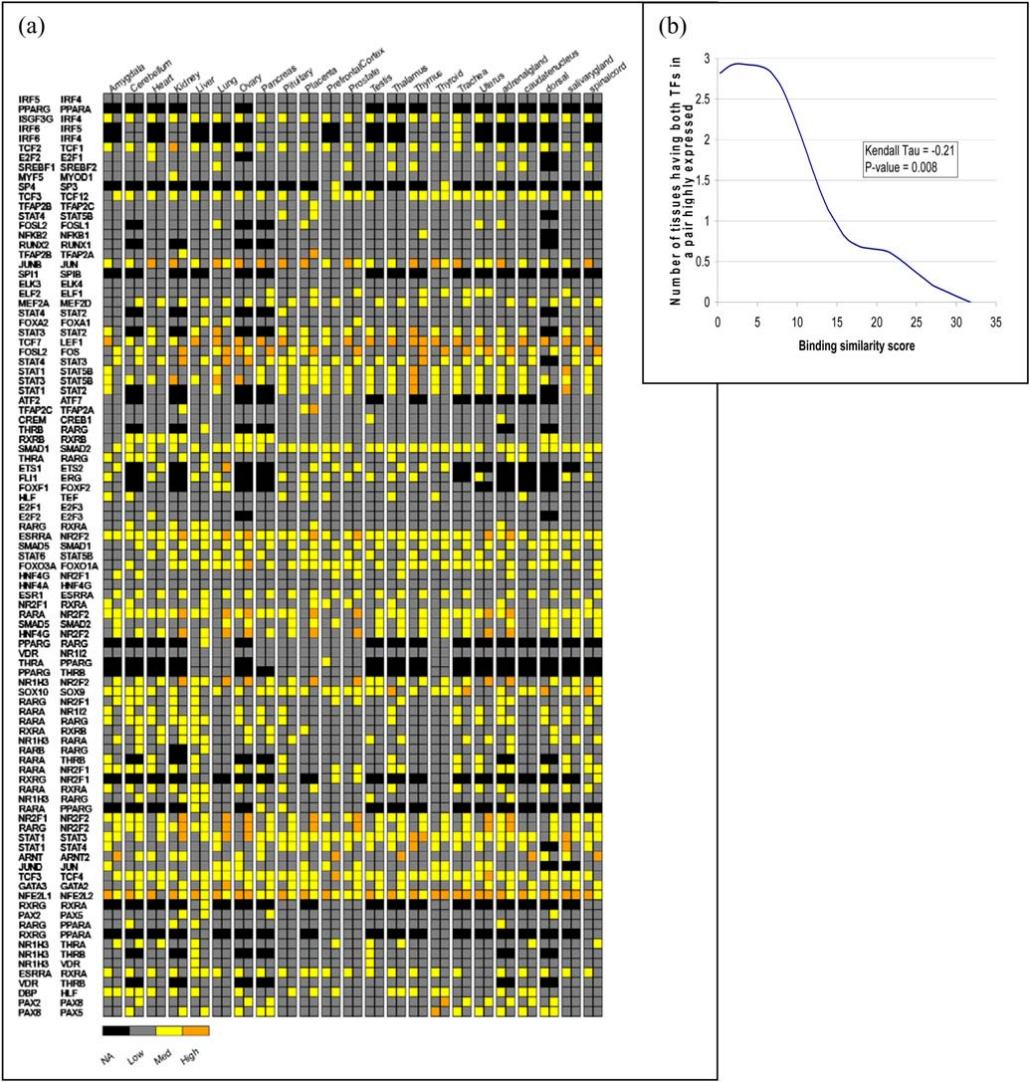
Expression of 95 pairs of TF paralogs in 23 non-redundant human tissues. (a) Columns correspond to the tissues and the rows correspond to the TF pairs. Rows are ordered in decreasing order of DNA binding similarity of TF pairs. The expression value for each TF is normalized in a tissue-specific way. The color of each cell represents the normalized expression level in each tissue for each TF in the pair. Grey cells indicate that the level of expression for this tissue was below our threshold for noise and hence, effectively zero. The thresholds are based on three equal splits of the range of expression values in a tissue-specific fashion. (b) Trend plot of the number of tissues where both TFs in a pair are highly expressed versus the DNA binding motif similarity score for the TF pair, using a cubic smoothing spline with 5 degrees of freedom. The (RXRB-RXRB) pair in fact corresponds to two distinct (partially overlapping) Ensembl genes. See Data file 2 for Ensembl gene ids.

We next sorted the 95 pairs of paralogous TF genes in decreasing order of their DNA motif similarity *§B*. Figure 5a exhibits a ‘heat plot’ of these 95 pairs of paralogous TFs; the columns indicate the 23 non-redundant tissues and the rows comprise the TF pairs. The expression value for each TF was normalized in a tissue-specific fashion by subtracting the median expression and dividing the result by the *Median Absolute Deviation* (*MAD*) statistic. The median absolute deviation for a data sample [25] is defined as *MAD* = *median*(|*Y_i_* – *median*(*Y_i_*)|), where |Y_i_| is the absolute value of Y_i_. The *MAD* statistic is preferred over the traditional z-score normalization (mean of 0 and standard deviation of 1) if the data is not normally distributed, because in the latter case the mean and standard deviation are severely affected by outliers[26]. The color of each cell represents the normalized expression level in each tissue for each TF in the pair. Black cells indicate that the level of expression for this tissue was below our threshold for noise and hence, effectively zero. The general trend observed in the section above is underscored by Figure 5 in that for the paralogous pairs with similar DNA binding site motifs (high *§B* towards the top of the list), there are very few cases where both paralogs are expressed at high levels, while this pattern is a relatively common occurrence among paralogs with low DNA binding similarity. At a glance, there is more homogeneity of high expression as the binding similarity of the TF pair decreases, reinforcing that the amount of expression divergence increases as the binding similarity increases. Figure 5b quantifies this trend by showing a plot of the number of tissues wherein both TFs in a pair are highly expressed as a function of the binding site motif similarity, using a cubic smoothing spline with 5 degrees of freedom. A cubic spline with 5 degrees of freedom essentially fits 5 piece-wise cubic polynomials over the data range to provide a smooth interpolation of the data. The resulting Kendall tau correlation between the numbers of tissues for which both TFs in a pair are highly expressed versus the binding similarity score is −0.21 with p-value 0.008. This plot indicates a clear inverse trend between the number of tissues for which both TFs in a pair are highly expressed and the binding similarity score. In Figure 5a, the expression values are discretized into three levels. In Figure 2 in supplementary Data File S1 the expression values are discretized into five levels, thus providing a finer grained depiction of the analysis.

### Age of paralogs

Because of the stringency of our criteria for paralogy, the TF pairs are likely to be recent paralogs. Therefore, we next investigated whether or not a greater DNA binding similarity is a simple reflection of shorter divergence time since duplication. In other words, it is expected that paralogs with similar DNA binding site motifs (high *§B*) are likely to be a result of recent duplication events and indeed if this were the case, the conservation in DNA binding site motif (and presumably in DNA binding domain of the gene) would merely be a reflection of divergence time. To test this hypothesis, for the entire set of TF paralogs we compared their BLAST-based percent identity score over the entire coding region (a high score is likely to correspond to recent duplication) with their *§B* values. The Kendal tau correlation was 0.063 with p-value = 0.36. We have repeated this analysis by substituting the BLAST-based percent identity with the synonymous substitution rate, Ks, using PAML [27] (see Methods), which yielded a consistent result with Tau = −0.067 and the p-value = 0.35. Thus, the observed relationships between *∂E* and *§B* are independent of the age of the paralogs. Figure 3 in supplementary Data File S1 demonstrates this result.

### Shared functions between paralogs

**Figure 6:**
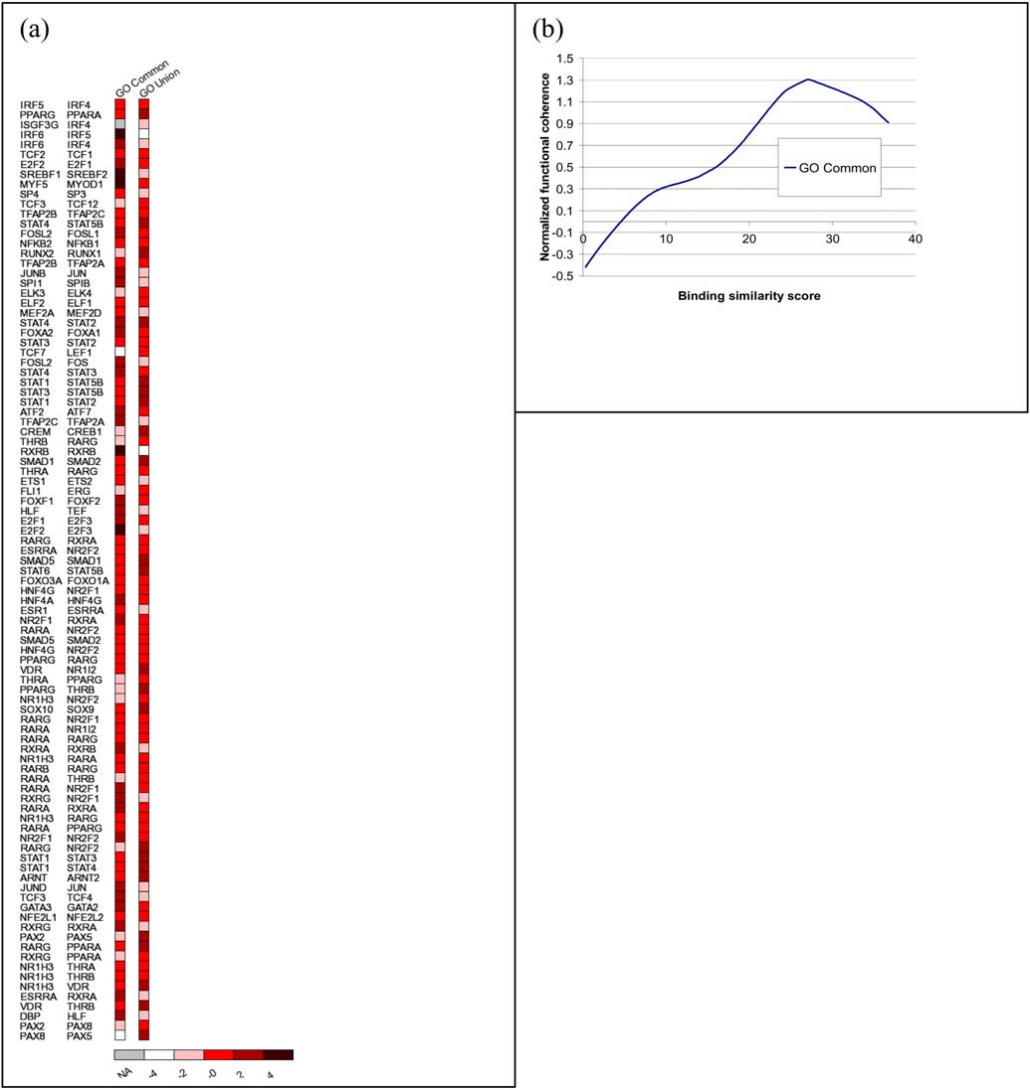
Functional coherence versus binding site motif similarity between TF paralogs. (a) The 95 paralogous TF pairs are sorted in decreasing order of DNA binding site motif similarity. Resulting values are normalized by subtracting the median value and dividing by the median absolute deviation. The normalized number of shared GO categories and the total number of GO categories are color coded. The corresponding figure legend indicates the normalized values. (b) The trend of normalized shared GO categories of paralogous TF pairs using a cubic smoothing spline with 4 degrees of freedom. Paralogs with high binding similarity tend to have more functions in common Kendall tau Correlation = 0.165, p-value = 0.02.

Next, we quantified the shared functions between paralogous TF pairs in order to determine if there is a correlation between DNA binding site motif similarity and biological function for a TF pair. For gene *X*, let *F*(*X*) represent the set of GO terms associated with *X*. We only consider terms at the functional hierarchy level of 3 or higher in order to exclude ubiquitous terms. For paralogs X and Y, we define *functional coherence* between the paralogs using the *Jaccard's coefficient* as *FC*(*X*,*Y*) = |(*F*(*X*) ∩ *F*(*Y*)| / |(*F*(*X*) ∪ *F*(*Y*)|, which represents the number of shared GO terms normalized by the total number GO terms annotated for the two genes *X* and *Y*. For the list of paralogs, sorted by decreasing order of *§B*, Figure 6a shows the heat plot and Figure 6b shows the trend plot of *FC*. We found that *FC* values were higher for TF pairs with higher binding similarity and gradually decreased as the binding similarity decreased. The Kendall tau correlation coefficient is 0.165 with p-value = 0.02. This indicates that paralogs with diverged DNA binding site motifs, and presumably different target genes, are more likely to serve distinct functional roles. Our results were consistent when we substituted the Jaccard's coefficient with a hypergeometric distribution based p-value to capture the overlap (Tau = −0.140, p-value = 0.04). This result further reinforces that paralogs with diverged DNA binding site motifs are more likely to serve distinct functional roles.

### Shared tissues between paralogs

**Figure 7:**
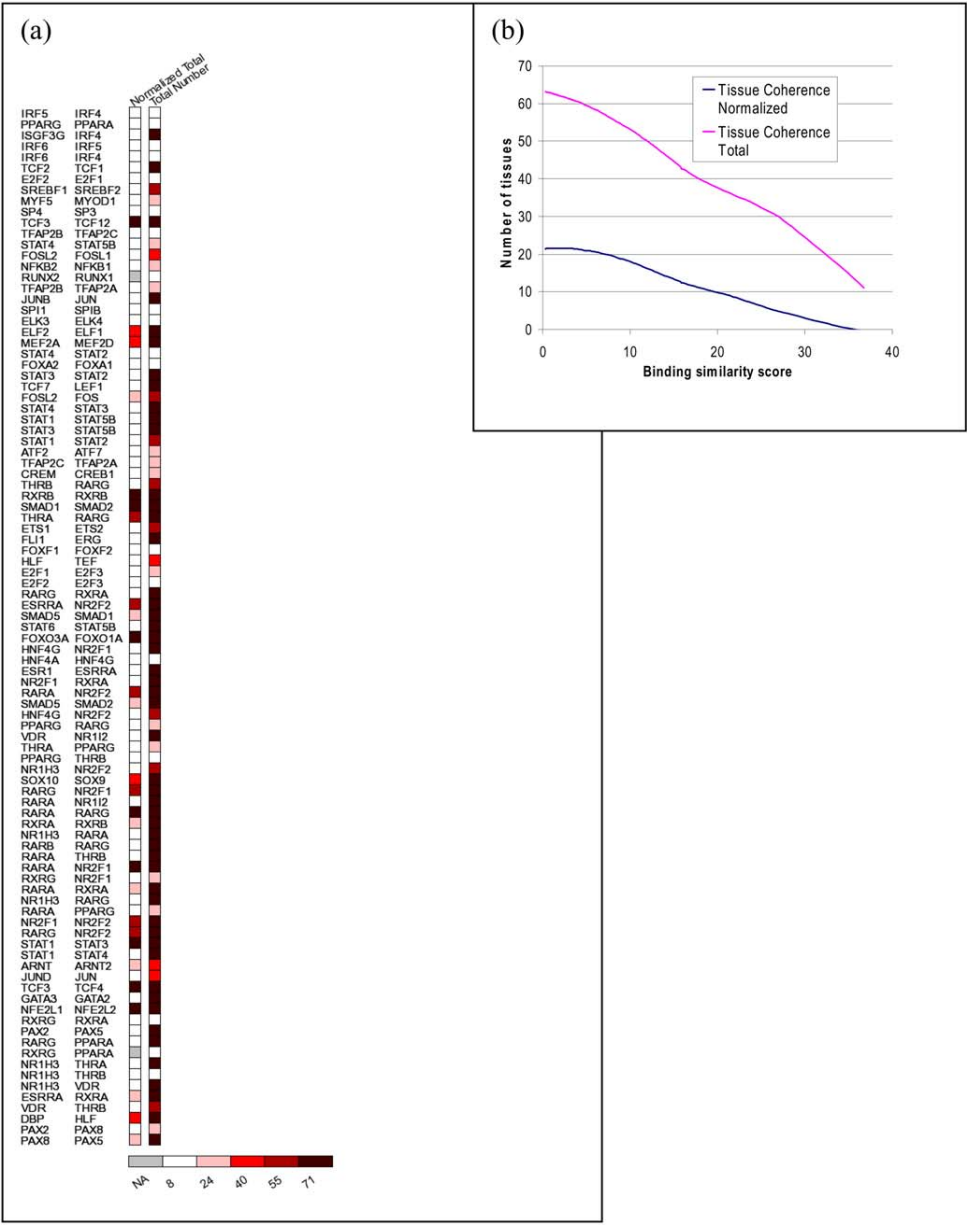
Tissue coherence versus binding site motif similarity between TF paralogs. (a) The 95 paralogous TF pairs are sorted in decreasing order of their DNA binding site motif similarity. The normalized and total number of shared tissues in which each TF is gene expressed at a level above the tissue-median are color coded, and the resulting figure legend indicates the number of tissues. (b) The trend of the normalized and total number of shared tissues for the paralogous TF pairs using a cubic smoothing spline with 4 degrees of freedom. Paralogs with high binding similarity tend to have few common tissues in which they are expressed, for both normalized and total. Kendall tau Correlation = −0.156, p-value = 0.03.

We quantified the shared tissues where paralogs are highly expressed and whether there is a correlation between the number of shared tissues and DNA binding site motif similarity. For paralogs X and Y, we defined *tissue coherence* between the paralogs as *TC*(*X*,*Y*) = |(*T*(*X*) ∩ *T*(*Y*)|/(*T*(*X*) ∪ *T*(*Y*)|. *T*(*X*) represents the set of tissues in which *X* is expressed above median expression value for that tissue. In the list of paralogs sorted by decreasing order of *§B* (Figure 7a), we found as expected from the above analysis, that *TC* values significantly decreased with increasing binding similarity. The Kendall tau correlation is −0.156 with p-value 0.03 (Figure 7b). In addition, the total number of tissues for which either of the TFs in a pair are significantly expressed also decreases as binding similarity decreases (Kendall tau correlation = −0.212, p-value = 3.7e-3). However, when we substituted the Jaccard's coefficient with the hypergeometric p-value, the correlation between the tissue overall and motif similarity is no longer significant. This is likely because the overlaps themselves are not significant in most cases (only in 12 of the 95 cases the overlap p-value≤0.05). Thus, while the tissue-overlap may not be significant, the degree of overlap is significantly correlated with the motif similarity. In other words, paralogs with similar DNA binding site motifs are expressed significantly less frequently within the same tissue at high levels, than paralogs with dissimilar DNA binding site motifs.

### Exclusive expression of one of the family members in a tissue

The above results suggest that among the paralogous TF pairs with similar DNA binding site motifs, at most one TF is expressed at a high level in any given tissue. Hence, we next extend our analysis to TF families. We computed families using two methods, the first is a complete-linkage agglomerative clustering of the 98 pair-wise paralogy relationships, which resulted in 39 paralog families with 28 families of size 2, 7 of size 3 and 4 of size 4; the rest are singletons which could not be placed in a family. We have repeated the family analysis for BLAST alignable coverage of 50%, 60%, 70% and 80%. The results are consistent for all four choices of alignable coverage, including criteria identical to that of Makova and Li, but we only report the statistics for the 70% coverage threshold. The second method is based on KOG data and in terms of families, we obtained 24 families, 9 of size 2, 6 of size 3, and 9 of size greater than or equal to 4. The family-wise analysis was done for (i) the 11 families consisting of greater than two TFs constructed using clustering methods and (ii) the 15 families computed using KOG data. For a TF family we expect at most one member of the family among the members with similar DNA binding site motif to have high expression in any tissue. In order to test this hypothesis, for each family with *k* TFs (*TF_1_*, *TF_2_*, ‥, *TF_k_*) and each tissue *T*, we computed the ratio *R* = (*E_max_*-*E_sim_*)/(*E_max_*-*E_min_*), where *E_max_* and *E_min_* represent the maximum and the minimum expression levels, respectively, among the family members in tissue *T*. Thus, the R value is normalized for family-specific and tissue-specific gene expression. If *TF_i_* has the maximum expression *E_max_*, then *E_sim_* is the expression level of the family member whose DNA binding site motif is most similar to that of *TF_i_*. This ratio effectively indicates how much the highest expressed gene in a family has diverged in expression relative to the gene with the most similar DNA binding site motif. Therefore, a ratio of *R* = 1 would indicate that the highest expressed gene in a family for a given tissue has most diverged in expression from the gene with which it shares the most binding similarity. In order to reduce the chances of considering irrelevant tissues (where all family members are expressed at low level), we only considered the tissues in which the maximum expression among the family members was greater than the median expression of all TF genes in that tissue. We then compared the *R* values computed from our dataset with that for 10000 random groupings of the TF genes in families, matched for family size of the actual dataset. Using the Mann-Whitney one-sided test we compared the *R* values of the foreground set with the random set given the null hypothesis that the *R* values in the foreground set are less than or equal to the *R* values in the random set. For the families constructed using clustering methods, the null hypothesis was rejected with a p-value = 6.84e-10. In the case of the families constructed using KOG data, the null hypothesis was not rejected when all families were used. However some of the families are very large (up to 16 members) and exclusion of large families yields significant results. For instance, when we only include families of size 3 and 4, the null hypothesis is rejected with a p-value = 1.3e-9. This result suggests that for all families with greater than two members and for all 23 non-redundant tissues, in general, exactly one family member is expressed highly, among the members with similar DNA binding site motif.

### Decrease versus increase in tissue-specific expression

We next investigated the relative prevalence of expression increase and expression decrease in a tissue among members of a paralogous family, irrespective of their DNA binding site motif similarities. Using the 11 families with greater than two members identified above, for each TF family and each tissue we enumerated the cases for which all but one family member have high expression and one member has low expression. High expression is defined as an expression value that is one *Median Absolute Deviation* or more than the median expression for that particular tissue. Low expression is the complement of high expression. We say that a family has a selective decrease in expression if all but one of the family members has high expression. Conversely, a family is deemed to have a selective increase in expression if all but one of the family members has low expression. We found that 38% of all cases were of expression increase and 20% cases of expression decrease. As a control, if we randomize the expression data, we found 48% of the cases were of expression increase and 16% were of expression decrease. The one-tailed Fisher exact test comparing decrease versus increase of expression for the true and random sets yields a p-value of 0.02, indicating that there is an excess of expression decrease in the actual data. If we repeat the analysis using families derived from KOG data, we find that 35% of the cases were of expression increase and 40% were of expression decrease. By comparison in the cases generated by randomizing the expression data, we found that 40% of the cases were of expression increase and 24% of the expression cases were of expression decrease. The one-tailed Fisher exact test comparing decrease versus increase of expression for the actual and random sets yields a p-value of 8.5e-7. This result further reinforces our findings that there is a significant tendency towards expression decrease in the families of TFs.

To further quantify the relationship of expression decrease versus expression increase we performed the following analysis. For each family of TF (*F_1_*, *F_2_*, ‥, *F_k_)* and each tissue *T*, we computed the ratio *RI* = (*E_max_*-*E_max2_*)/(*E_max_*-*E_min_*), where *E_max_* and *E_min_* represent the maximum and the minimum expression levels and *E_max2_* represents the second highest expression among the family members in tissue *T*. The ratio *RI* captures the increase in expression, i.e., high value of *RG* represents an expression increase. Similarly, define *RD* = (*E_min2_*-*E_min_*)/(*E_max_*-*E_min_*), where *E_min2_* represents the second lowest expression among the family members in tissue *T*. The ratio *RD* captures decrease in expression, i.e., high value of *RL* represents an expression decrease. We computed *RI* and *RD* values for all TF family-tissue pairs and did the same for 10000 randomly generated families of TFs of the same size as the true set of families for families defined using the BLAST criterion and families defined using KOG data. We found that the *RI* values (increase) in the actual data were significantly smaller than those for the randomized families (Mann-Whitney one-sided test p-value = 0.0023 for paralogs defined using BLAST criterion and p-value = 5.2e-4 for the families defined using KOG data). In contrast, the *RD* values (decrease) in the actual data were greater than those for the randomized families. However, the Mann-Whitney one-sided test p-value was at the cusp of significance (p-value = 0.06) for families defined using BLAST criterion. For families defined using KOG data, we found that RD values (decrease) were significantly less than those for randomized families (Mann-Whitney, one-sided test p-value = 3.4e-8. Thus, it appears that relative to background expectation, a decrease in expression appears to be more common and of a greater magnitude than an increase in expression in gene families.

## Discussion

Gene duplication followed by functional diversification of the duplicated genes is a major driver of evolution[1]–[4]. However, there are evolutionary scenarios where duplicated genes are maintained if increased dosage is advantageous, e.g. in the case of immunity related genes[12], [13]. Gene paralogs may functionally diversify through mutations in their coding sequences, thereby changing the activity of the gene product, or through mutations in the regulatory sequences, thereby altering the gene expression[28], or through both processes. The pathways of functional diversification and their interrelationships are of fundamental interest from the perspective of understanding adaptive expansion of gene families[2], [29], [30]. While mutations may alter either the coding or the regulatory sequence of a gene, it is the resulting functional change – in expression or in protein activity – that is likely to determine the gene's evolutionary fate. Although it is often not possible to quantify the functional consequence of a mutation, TF genes offer a unique opportunity in this respect. By explicitly quantifying one aspect of functional diversification, viz., DNA binding site motifs, we have demonstrated that paralogs with conserved DNA binding site motifs tend to diverge in their tissue-specific expression and conversely, paralogs that are highly expressed in a tissue tend to have dissimilar DNA binding site motifs and thus, different target genes. Although we have observed an overall significant trend, the inverse relationship between the two modes of diversification is likely to apply to a subset of paralogs, and several other evolutionary processes are likely to be active.

We have taken a number of precautions to ensure the robustness of our conclusions. For instance, Pearson's correlation coefficient is a standard measure for quantifying correlations between two data samples. However, PCC is applicable only when the data are normally-distributed. Consequently, we have chosen the more appropriate but conservative Kendall's tau to measure correlation between the tissue-specific expression divergence (*∂E*) and DNA binding site motif similarity (*§B*). We preferred Kendall's tau to another non-parametric correlation measure – Spearman's rho, because Kendall's tau has better statistical properties and is more directly interpretable [31] (see methods). However, using either Spearman's rho or PCC does not affect our results appreciably and does not affect our conclusions. Also, because multiple paralogous pairs involve the same TF gene, the individual *∂E* and *§B* values cannot be assumed to be independent. To avoid biases caused by within-sample dependence, we estimated the significance of Kendall's tau by randomly permuting the expression values among the TFs and our results remain statistically significant. Thus, our overall results remain significant across a comprehensive set of combinations of experimental design parameters - (i) multiple ways of probe aggregation for a gene or a transcript (ii) multiple PWM aggregation for a gene or a transcript, (iii) multiple measures of correlation, and (iv) multiple ways of estimating significance.

Prior investigations of functional diversification among paralogs have only considered sequence divergence in the entire coding portion of the gene[2], [29], [30]. Makova and Li found a negative correlation between expression divergence and protein sequence divergence in human paralogs[21]. To quantify expression divergence between paralogs, the prior works have used Pearson's correlation coefficient or Euclidian distance (Jordan, et al 2005) using expression profiles of two genes across multiple contexts (tissues in case of Makova and Li). However, most genes, particularly the TF genes, are likely to be functionally relevant only in a few tissues or experimental conditions[32]. Thus, a context-specific analysis of expression divergence is more informative. Our finding in human is consistent with that of Makova and Li even though we measure expression divergence (*∂E*) in a context-specific manner. In fact when we replace tissue-specific *∂E* value with an overall measure of expression divergence using Pearson's correlation coefficient between the expression profiles across the 79 (or 23) tissues, we do not observe a significant correlation with DNA binding site motif similarity as shown in Figure 1 in supplementary Data file S1. This phenomenon is not surprising because it is the absolute expression level in specific contexts that is relevant to evolution. While it is well-established that duplicated genes have undergone rapid divergence in expression immediately after duplication[29], our work refines this general observation by showing that the expression divergence may be influenced by divergence in other aspects of gene's function, for instance, DNA binding site motif of the TF protein.

Given the multitude and complexity of attributes affecting evolution, most of which are not completely understood, we expect that most individual correlations will be weak. Strong correlations are indeed scarce in biological literature. Liao et al. studied the correlations between evolutionary rates of mouse paralogs and several genic parameters and remarked that “*because there are potentially many rate determinants …… it is not unexpected that the observed correlation coefficients are not very high*”[33]. There are some concerns with using the expression data in investigating functional diversification, or indeed in any functional study. First, the expression level serves only as a proxy for the amount of active transcription factor protein. However, an accurate genome-wide quantification of active transcription factors in various tissues is currently not feasible. Another concern is that when two paralogs have very similar expression, their corresponding probes on the microarray are likely to cross-hybridize, thus confounding the results. However, the possibility of a cross-hybridization serves to make our finding more conservative.

Expression pattern and the DNA binding site motif represent two distinct aspects of TF function. There are others, such as interaction partners, and these distinct functional aspects are often encoded in distinct sequence domains. For instance, the DNA binding site motif is encoded largely within the DNA binding domain of the gene. In the human Forkhead (FKH) domain containing family of TFs, the sequence similarity in DNA binding FKH domain is significantly correlated with the DNA binding site motif similarity (R = 0.47, P = 9.6e-05; data not shown). Because selection pressure operates at the level of function, which is encoded in distinct domains, it is reasonable to study the relationship between diversification in distinct domains. Again, in the human FKH family, where the DNA binding domain is highly conserved across family members, the rest of the protein domains exhibit an accelerated divergence relative to the DNA binding domain immediately after duplication (data not shown). A specific comparison of divergence in the TF's interaction domain with either the divergence in DNA binding similarity or the expression divergence or both, would be a natural extension of the current work. However, the interaction domains of TF proteins are currently not as well characterized as the DNA binding domains. As a proxy for divergence in interaction domain, one can consider the overlap in known interaction partners. However, such an investigation is currently limited by the availability of complete interaction data. Our work generalizes the previous studies of diversification of paralogs that have focused on divergence in protein coding sequence in its entirety without distinguishing among functional protein domains and without quantifying the functional consequence of mutations in the coding sequence.

As a pragmatic concern, current genomic approaches to analyzing transcriptional regulation are confounded by the fact that multiple TFs, typically closely related paralogs, bind to similar binding sites. For instance, previous analysis of motifs enriched in promoters of genes that are differentially expressed in adult failing hearts identified the FKH family. However, to implicate specific members of the Forkhead family of TFs, directed experiments such as PCR and immunohistochemistry in specific cell types are needed[34]. Our results here not only explain in part the apparent redundancy in the DNA binding specificity of transcription factors – TFs with similar binding have dissimilar expression pattern – but also underscore the importance of incorporating the expression of the transcription factor genes in the analysis of transcriptional regulation.

## Materials and Methods

### TF proteins and their DNA binding specificities

A total of 390 distinct human TFs were obtained from TRANSFAC 10.2[35]. Using the annotation table provided by TRANSFAC, PWM identifiers were mapped to 297 unique UniProt accession identifiers. These UniProt identifiers were then mapped to 295 unique Ensembl gene identifiers using Ensembl-47 (www.ensembl.org). For each of the TFs, the corresponding positional weight matrix (PWM) representing their DNA binding site motif was obtained from TRANSFAC.

### Identifying paralogous protein pairs

As in several previous studies, a BLAST-based approach was used as one of the possible methods for determining paralogy[36]. BLAST was performed by querying human TF protein sequences from a database of all human protein sequences obtained from Ensembl-47. Two TFs were deemed to be paralogous if (i) the BLAST E value≤1.0e-5, (ii) the BLAST-alignable region is ≥70% of the longer protein or the length of the longest High Scoring Pair (HSP) is >150 aa, and (iii) the identity (I) is ≥ 5% if the alignable region is longer than 150 aa or *I*≥0.06*n*+4.8*L* ^−0.32[1+exp(−*L*/1000)]^ where *L* is the length of alignable region. A similar set of criteria was used in [21]. One difference is that Makova and Li use 80% as the threshold for the coverage of alignable region while we use 70%. In addition, we also use a more stringent E value for identifying significant HSPs (E≤1.0e-5 vs. E≤1 in Makova nd Li). Based on these criteria we identified 95 paralogous TF gene pairs. Our criteria for paralogs, as for the previous works, are stringent and detect relatively recent paralogs. There are two difficulties in analyzing distant paralogs. First, inference of paralogy will be less accurate and second, the divergence in expression and binding site motifs are likely to have reached saturation and thus, we do not expect to detect a significant correlation between the two quantities. In fact our criteria is slightly less stringent that that of Makova and Li. When we use identical criteria, despite a reduction in the number of paralogous pairs, the results do not change appreciably.

### Similarity in DNA binding site motif for TF pairs

The likelihood of two DNA binding site motifs, or PWMs, having identical DNA sites can be approximated using a number of pair-wise PWM similarity measures. Mahoney et al. have reported a detailed benchmarking study of several measures of PWM-PWM similarity[23]. We utilize the best performing Pearson correlation (PCC) based measure (provided by Shaun Mahoney). Very briefly, this particular method measures the similarity between two columns of the two PWMs using the Pearson correlation coefficient. Then using the PCC as the column ‘match’ score, the method aligns the two PWMs using the traditional Smith-Waterman algorithm for sequence alignment. The median pair-wise PWM similarity scores for the 95 TF-pairs used in the main analysis are provided in worksheet #1 in supplementary Data file S2. We have additionally ensured that the pairwise motif similarity is not correlated with the motif complexity (Data file S1).

### Gene expression

Human gene expression data was obtained from the Novartis human tissue survey [20] processed with the gcRMA preprocessing probe set algorithm and consists of 79 different tissue samples. We used this data directly provided “as is” from the Novartis website without any further processing steps. We refer the readers to their web documentation (wombat.gnf.org) for details. However, we only considered data from the U133A human array since we had reliable annotation data only for this chip. The gcRMA processed data was chosen since gcRMA offers a good balance between accuracy and precision for analyzing gene expression data, and correcting for background noise[37]. We also repeated our analysis on a subset of 23 tissues that were previously identified to be independent and thus, non-redundant[24]. Each sample included expression values for 17220 human genes. Recently, there have been several papers addressing the interpretation of Affymetrix gene expression data[38], [39]. We have chosen the mappings from probe to transcript protein mapping (IPI identifier) provided in [39] for the U133A human array in our analysis. Mappings from IPI protein identifier to genes were obtained from Ensembl release 47 (www.ensembl.org). Because multiple probes are mapped to a single gene or transcript, the probe-level data needs to be aggregated in order to obtain a gene-level or transcript-level expression value, which invariably raises issues of accuracy. Probe-level expression analysis bypasses these issues and yield reliable results[39]. However, we have shown that our primary result holds at the probe, transcript and gene level, with three different approaches to aggregating the probe-level data (see below). It is also well-established that the lowest level intensity probes are most prone to error. Therefore, we have disregarded all probes having a value less than 50 (∼8% of all probes). The median gene expression values for the 98 TF genes used in this study across 79 tissues are provided in worksheet #2 in supplementary Data file S2.

### Genes and transcripts having multiple probe sets

On the Affymetrix chip underlying the genome-wide expression studies, genes are often represented by multiple probe sets and thus, have multiple expression values. Several solutions have been posed to contend with this issue: (i) only choosing probe sets with a one-to-one correspondence with genes[40], (ii) choosing for each gene the probe set with the maximum expression [41] and (iii) choosing for each gene a probe set at random[42]. To account for the expression values for all probe sets while minimizing the effect of erroneous values, as the default, we used the median expression value across the probe sets for a given gene. However, to ensure the robustness of our results, we have repeated our analysis by also using both the maximum probe value and selecting one probe set at random given a choice of multiple probe sets. Similarly, we have used three aggregation strategies for the transcript-level analyses and finally we have also done a probe-level analysis to bypass the aggregation altogether.

### Kendall tau measure of correlation

Pearson correlation coefficient (PCC) is a standard measure of interdependence between two random variables. PCC is applicable only when we wish to measure a linear relationship between two variables and the variables are from bivariate normal distributions. Because we cannot assume normally-distributed data in our case, we require a nonparametric measure of correlation. The two most common choices are Spearman's rank order correlation (Spearman rho) and Kendall tau correlation. Both measures offer similar sensitivity in detecting associations and almost always lead to the same conclusions. However, we use Kendall's tau because it has better statistical properties and there is a direct interpretation of Kendall's tau in terms of probabilities of observing concordant and discordant pairs[31]. It is important to note that the correlation measure we have employed is inherently conservative. As a result, we do not expect strong correlation values based on the nature of the data being dealt with. The more relevant factors for our analysis are the significance and sign of correlations.

Our choice of correlation measure inherently assumes that the input data consists of independent samples. In our application, this assumption is not necessarily true and consequently employing the theoretical p-value estimation of Kendall' tau is potentially erroneous. Therefore, we chose to use a permutation-based method for computing the p-value for Kendall's tau. In a given tissue sample, we pool all expression values for all TFs in our dataset, and then randomly assign expression values to each TF. This procedure effectively shuffles the TF expression values and hence shuffles the corresponding *∂E* values for the paralogous TF pairs. We compute the Kendall's tau for 1000 such permutations and use the fraction of permutations in which the tau value exceeds the observed tau value (for the un-shuffled data) as an estimate of the p-value.

### KOG family analysis

KOG data and the associated gi identifiers were obtained from ftp://ftp.ncbi.nih.gov/pub/COG/KOG/. The proteins were mapped to Ensembl transcript and gene ids using data from www.ensembl.org. The KOG data resulted in 242 paralogous pairs with 99 unique genes. In terms of families, we obtained 24 families, 9 of size 2, 6 of size 3, and 9 of size greater than or equal to 4.

### Ks analysis

Nucleotide coding sequences were obtained from Ensembl for all TFs, and the pairwise Ks rates computed using the bp_pairwise_kaks.pl script from BioPerl[43].

## Supporting Information

Data File S1 This file contains supplementary results
(0.22 MB DOC)

Data File S2 This contains supplementary data in two worksheets
(0.04 MB XLS)
