# Supplementary material for: Functional Diversification of Paralogous Transcription Factors via Divergence in DNA Binding Site Motif and in Expression
Source: PLoS One. 2008 Jun 4;3(6):e2345. doi: 10.1371/journal.pone.0002345 (PMC2394658; doi:10.1371/journal.pone.0002345)
Supplement: Data File S1 — This file contains supplementary results (0.22 MB DOC) [file pone.0002345.s001.doc]

***Functional diversification of paralogous transcription factors via divergence in DNA binding site motif and in expression***

Authors: Larry N. Singh and Sridhar Hannenhalli

**Correlation between *∂E* and *§B* is significant even when we exclude the paralogous pairs with low expressions**

To ensure that the observed correlation between *∂E* and *§B* is not mainly due to the paralogous pairs where both paralogs have low expression, we repeated the above experiment after removing all TF pairs for which the expression levels of both TF genes in the pair were in the bottom 25^th^ percentile. We found a significant correlation in 14(61%) of the 23 tissues. We repeated this analysis after removing all TF pairs for which the expression levels of both TF genes in the pair were in the bottom 33.3^rd^ percentile.  In this case, of the 23 tissues, 11(48%) showed significant correlation. We believe that the reduction in the number of tissues that reveal significant correlation is in part because of the reduction in sample size.  Notwithstanding, the results remain significant even after removing all TF gene pairs for which both genes in the pair had low expression in a tissue.

We also measured the correlation between binding similarity and more traditional measures of expression divergence, in particular divergence measured as *abs(E_X_-E_Y_)*.  This measure however over-interprets the absolute expression and does not capture the qualitative differences in expression, e.g. low-high versus high-high.  Consider two pairs *(u1 v1)* and *(u2 v2)* and let the expression of *u1 = x*, expression of *v1* and *u2 = (x+y)* and the expression of *v2 = (x+2y)*, for arbitrary value of *x* and *y*. In this case the absolute expression difference is the same for the two pairs – *y*, which does not capture the fact the both *u2* and *v2* may be “highly” expressed while in the first pair, *u1* may be lowly expressed (depending on *x*) while *v1* is highly expressed (depending on *y*). The inverse-harmonic-mean measure that we use makes an appropriate distinction between these cases. When we repeat the analysis with *abs(Ex-Ey)*, we do see a significant correlation in 47.8% of the tissues, but only after we exclude TF pair with both genes expressing below 66.7 percentile of all expression values in that tissue.

**Correlation between *∂E* and *§B* is robust to alternative methods of aggregating multiple probe data and multiple PWMs**

For the analysis presented above, if multiple probe sets mapped to the same gene, we used the median expression value of the probe sets as the gene’s expression. To ensure the robustness of our results, we also considered as alternatives, the maximum expression value and a random expression value among the probe sets that mapped to a gene. Similarly, if a particular TF gene corresponded to multiple PWMs in TRANSFAC, we considered both the median of all pair-wise PWM similarities and similarity between randomly selected PWMs. In addition to the gene-level analysis, we also performed the analysis at the level of transcripts and probes. Thus, we repeated the entire correlation analysis with 14 different combinations – three choices of probe aggregation (median, max, random) for genes and transcripts, plus the probe level analysis, and for each of these 7 scenarios we used two choices of PWM aggregation (median and random). We performed the correlation analysis for these 14 scenarios and the fraction of 23 tissues that show significant positive correlation between *∂E* and *§B* are listed in Table 1. On average, across these 14 experiments, 86% of tissues show significant correlation between *∂E* and *§B*. Thus, our overall finding is robust and hence, indicative of a true evolutionary trend.

**Motif similarity versus motif complexity**

We tested whether the motif similarity score is related to motif complexity and we found this not to be the case. For instance, motif AAAAAAA has very low complexity while the motif ACCCTTG has high complexity. We measured the *complexity* of a motif as the product of base frequencies in the motif consensus after adding a pseudo-count of 0.1. We used both, minimum and the average complexities of the two motifs to be the complexity of the pair. The Kendall tau correlation between motif-pair complexity and the motif-pair similarity was -0.005 (using average complexity) and -0.01 (using minimum complexity), both of which are insignificant. Thus, we found that motif-pair similarity is independent of the motif complexity.

**Table 1: Summary of correlation analysis for 14 combinations of experimental parameters.** Summary of the number of tissues for which there is significant correlation between *∂E* and *§B* for various measures of expression and binding similarity aggregate scores. Expected fraction of tissues showing significant correlation (p-value ≤ 0.05) is 5% as shown based on randomized data (see text).

| **Binding Similarity Aggregate Measure** | **Probe** | **Transcript** | | | **Gene** | | |
| --- | --- | --- | --- | --- | --- | --- | --- |
|  |  | **Median** | **Max** | **Random** | **Median** | **Max** | **Random** |
| **Median** | 23(100%) | 18(78%) | 17(74%) | 19(83%) | 17(74%) | 14(61%) | 18(78%) |
| **Random** | 23(100%) | 23(100%) | 23(100%) | 23(100%) | 20(87%) | 19(83%) | 20(87%) |

**Supplementary Figure 1**. Scatter plot showing relationship between DNA binding site motif similarity between paralogous TF pairs and the Pearson expression correlation across 23 tissues for the TF paralogous genes.

| | 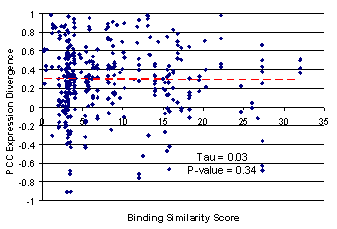 | | --- | |
| --- | --- |

**Supplementary Figure 2**. Same as Figure 5 but using 5 levels of discretization to present a more refined depiction of the expression divergence among paralogs. The thresholds are based on equal splits of the range of expression values in a tissue-specific fashion.

| | 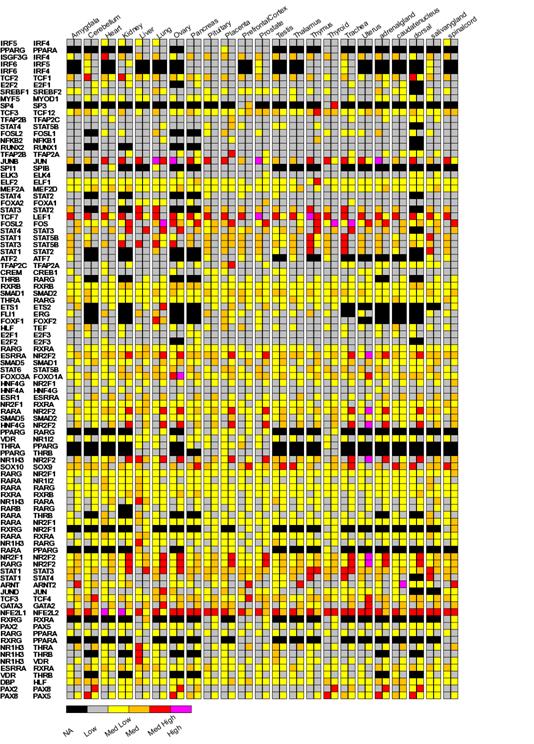 | | --- | |
| --- | --- |

**Supplementary Figure 3. Sequence similarity versus DNA binding site motif similarity between TF paralogs.** (a)The 95 paralogous TF pairs are sorted in decreasing order of their DNA binding site motif similarity. The BLAST sequence similarity is color coded, and the corresponding figure legend indicates the percentage identity. (b) The trend of the relative age of the paralogous TF pairs using a cubic smoothing spline with 10 degrees of freedom.  The trend line within the plot is relatively flat (Kendall tau Correlation = 0.063 p-value = 0.37) indicating little or no correlation between the two quantities.

| 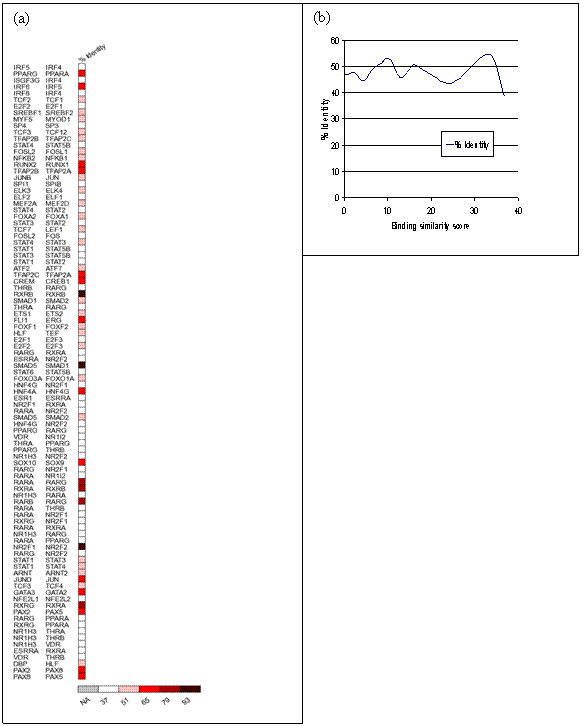 |
| --- |
